# The problem lies in the medium: challenges for diagnostic use of spent culture media in IVF

**DOI:** 10.3389/frph.2026.1791226

**Published:** 2026-04-01

**Authors:** Zuzana Holubcová

**Affiliations:** 1 Department of Histology and Embryology, Faculty of Medicine, Masaryk University, Brno, Czechia; 2 Reprofit International, Clinic of Reproductive Medicine and Gynecology, Brno, Czechia

**Keywords:** assisted reproduction, biomarkers, embryo selection, *in vitro* fertilization, non-invasive diagnostics, quality control, spent culture media

## Introduction

Analysis of spent embryo culture media (SCM) has been widely explored as a source of molecular biomarkers reflecting embryo viability and implantation competence. Yet after more than two decades of research, no SCM-based biomarkers have been translated into routine clinical use. This opinion article argues that this persistent lack of translation reflects fundamental methodological constraints rooted in the historical evolution of embryo culture media. Limited compositional transparency, batch-to-batch variability, and protein supplementation introducing uncontrolled background signals compromise the reproducibility of SCM studies and confound molecular readouts. Because the culture medium itself is not well defined, signals detected in SCM cannot be confidently attributed to the embryo, making biomarker validation extremely challenging. By highlighting these issues, this article calls for critical debate on standardization, transparency, and quality control in embryo culture media production.

## The evolution of IVF culture media

Early human embryos produced by *in vitro* fertilization (IVF) develop within culture systems designed to support survival and progression to the blastocyst stage. The earliest culture media consisted of simple salt solutions intended to preserve basic cellular viability, but these proved insufficient to sustain robust preimplantation development (1, 2). As understanding of embryo physiology advanced, media compositions expanded to include carbohydrates, serum components, and buffering systems (3, 4). Continued efforts to improve culture performance led to progressively more complex formulations incorporating amino acids, antioxidants, vitamins, antibiotics, chelators, and macromolecular supplements (5–8). Most of these components were introduced empirically, with limited systematic characterization of their individual or combined effects. Collectively, these developments enabled routine extended culture to the blastocyst stage and contributed to better *in vitro* developmental outcomes (9).

In parallel with these advances, media production shifted from in-house laboratory preparation to large-scale commercial manufacturing. Centralized production improved end-user convenience, reduced operator-dependent variability, and facilitated the global implementation of standardized laboratory workflows, but it also reduced transparency regarding media composition. Commercial IVF media are typically proprietary formulations, with manufacturers disclosing only partial compositional information, often citing intellectual property protection. This historical trajectory has left a legacy that complicates reproducibility, limits biological interpretation, and poses challenges for downstream analytical applications.

## Spent culture media as a diagnostic readout: promise meets reality

In addition to their role in supporting embryo development, IVF culture media have increasingly been repurposed as sources of diagnostic information. The concept of analyzing SCM as a source of non-invasive biomarkers is intuitively appealing. During culture, the embryo consumes nutrients and releases metabolites and signaling factors into its surrounding environment (9). Advances in omics technologies now allow detection of these changes from minute sample volumes, raising the prospect that embryo assessment could be informed by objective, non-invasive molecular criteria rather than relying solely on subjective morphology or requiring invasive embryo biopsy (10–12).

Over the past two decades, numerous studies have reported associations between specific molecular signatures and embryo morphology, ploidy status, or implantation outcome (13–18). However, despite sustained effort and continuous technological advances, no SCM-derived biomarkers have been validated for routine clinical use. This persistent lack of clinical translation can be attributed to poor reproducibility of published data. Systematic reviews highlight that reported findings vary widely between studies, reflecting differences in culture conditions, sampling time points, analytical platforms, outcome definitions, and data reporting practice (19–21). Under such conditions, results cannot be reliably confirmed across independent datasets.

Beyond differences in study design and analytical approaches, multiple physical and biological aspects of the embryo culture environment are known to influence embryonic metabolism and stress responses and therefore may affect SCM-derived molecular readouts. These include oxygen concentration, pH stability, temperature control, oil overlay properties, light exposure, and the presence or absence of paracrine signaling within the culture system (22, 23). These factors undoubtedly contribute to inter-study heterogeneity and require careful control at the laboratory level.

By contrast, a central but insufficiently acknowledged limitation of SCM analysis is that the culture medium itself is variable and incompletely characterized. Analytical studies have repeatedly demonstrated substantial variability between commercial brands and lot-to-lot differences within the same product (24–29). This lack of standardization undermines the reproducibility of culture conditions and complicates comparisons of both clinical outcomes and research findings across laboratories. Importantly, this compositional variability is not merely a technical concern. Clinical studies report associations between embryo culture media and IVF outcomes underscoring that variation in culture conditions can have clinically meaningful consequences (30–34). Experimental evidence further indicates that embryonic metabolism can modulate epigenetic states (35–37), suggesting that the availability of specific nutrients and metabolic intermediates may influence cell fate decisions and overall developmental competence.

From an analytical perspective, differences in blank media composition, including batch-to-batch variation, confound interpretation of SCM-derived signals by shifting the molecular baseline against which embryonic changes are measured. As a result, molecular features attributed to the embryo may instead reflect differences in starting conditions rather than true embryo-derived biological variation.

This problem is particularly evident at the protein level. Serum-derived albumin is routinely added to culture media to support development (38). However, this blood-derived carrier protein retains plasma-origin molecules that vary between batches and introduce a complex and unstable background into the culture system (29, 39–43). Some of the proteins co-purified during manufacturing can act as growth factors or hormones (29) and may therefore contribute positively to embryo development. At the same time, the protein background introduced by human serum albumin supplementation compromises culture reproducibility and complicates interpretation of signals detected in SCM, making it difficult to distinguish embryo-derived factors from albumin-associated contaminants (29, 43).

Taken together, these limitations indicate that the persistent lack of clinically useful SCM-based biomarkers does not primarily reflect an absence of biologically meaningful signals, insufficient analytical sensitivity or inadequate sample size. Rather, it represents a system-level limitation arising from how IVF culture media are designed and used. Culture media were optimized to support embryo development, not to provide a stable and interpretable molecular baseline. Because molecular diagnostics require reproducible baseline conditions, the insufficient standardization and transparency of the IVF culture environment create a fundamental mismatch between diagnostic ambition and infrastructural design. Without greater control over the composition and consistency of the culture environment, the diagnostic potential of SCM remains constrained.

## Conclusion: transparency and standardization as prerequisites for SCM diagnostics

SCM analysis offers a window into the molecular state of the preimplantation human embryo. However, realizing its diagnostic potential requires stabilizing the frame through which this biology is observed. Current embryo culture media, shaped by decades of empirical optimization for developmental support, are chemically complex and incompletely defined and are therefore inherently unsuited for reproducible molecular readouts. Under current conditions, SCM analysis is not impossible, but it demands a level of experimental control, baseline definition, and documentation that substantially exceeds what is currently standard in research studies. As long as the molecular baseline remains variable, even biologically meaningful embryo-derived signals will remain difficult to resolve.

Progress in SCM-based diagnostics will therefore depend less on further analytical refinement and more on a conceptual shift in how culture media are manufactured and regulated. A necessary first step is greater transparency regarding the composition of commercial culture media and the variability introduced by manufacturing processes. Transitioning toward chemically defined formulations free from blood-derived components would eliminate a major source of variability, allowing genuine biological signals to be distinguished from artifacts and enabling systematic evaluation of how specific molecular factors influence embryo development.

Deeper understanding and genuine standardization of media composition are matters of clinical responsibility. The IVF community must take an active role in establishing clear standards for media formulation and rigorous quality control strategies, both to support evidence-based clinical practice and to unlock the diagnostic potential of SCM analysis.
